# Genetic Adaptations of an Island Pit-Viper to a Unique Sedentary Life with Extreme Seasonal Food Availability

**DOI:** 10.1534/g3.120.401101

**Published:** 2020-03-17

**Authors:** Bin Lu, Xiaoping Wang, Jinzhong Fu, Jingsong Shi, Yayong Wu, Yin Qi

**Affiliations:** * Chengdu Institute of Biology, Chinese Academy of Sciences, Chengdu 610041, China; † Nature Conservation of Snake Island and Laotieshan Mountain, Dalian 116041, China; ‡ Department of Integrative Biology, University of Guelph, Guelph, Ontario N1G 2W1, Canada; § Key Laboratory of Vertebrate Evolution and Human Origins of Chinese Academy of Sciences, Institute of Vertebrate Paleontology and Paleoanthropology, Chinese Academy of Science, Beijing 100044, China; ** University of Chinese Academy of Sciences, Beijing 100049, China; †† College of life sciences and food engineering, Yibin University, Yibin 644007, China

**Keywords:** Genetic adaptations, transcriptome, extreme sedentary life, *Gloydius shedaoensis*

## Abstract

The Shedao pit-viper (*Gloydius shedaoensis*) exhibits an extreme sedentary lifestyle. The island species exclusively feeds on migratory birds during migratory seasons and experiences prolonged hibernation and aestivation period each year (up to eight months). The sedentary strategy reduces energy expenditure, but may trigger a series of adverse effects and the snakes have likely evolved genetic modifications to alleviate these effects. To investigate the genetic adaptations, we sequenced and compared the transcriptomes of the Shedao pit-viper and its closest mainland relative, the black eyebrow pit-viper (*G. intermedius*). The Shedao pit-viper revealed a low rate of molecular evolution compared to its mainland relative, which is possibly associated with metabolic suppression. Signals of positive selection were detected in two genes related to antithrombin (*SERPINC1*) and muscle atrophy (*AARS*). Those genes exert significant functions in thrombosis, inhibiting oxidation and prolonged fasting. Convergent and parallel substitutions of amino acid with two other sedentary vertebrates, which often suggest adaptation, were found in a fatty acid beta-oxidation related gene (*ACATA1*) and a circadian link gene (*KLF10*), which regulate lipogenesis, gluconeogenesis, and glycolysis. Furthermore, a circadian clock gene (*CRY2*) exhibited two amino acid substitutions specific to the Shedao pit-viper and one variant was predicted to affect protein function. Modifications of these genes and their related functions may have contributed to the survival of this island snake species with a sedentary lifestyle and extreme seasonal food availability. Our study demonstrated several important clues for future research on physiological and other phenotypic adaptation.

Organisms often face challenges from strong seasonality, where key climatic variables and resources (*e.g.*, temperature, rainfall, food availability) may vary drastically from season to season, and many have evolved strategies to survive such seasonal changes. Prolonged immobilization including hibernation and aestivation are commonly employed by many animal species (Hiong *et al.* 2013; Bose *et al.* 2016; Faherty *et al.* 2016). Inactivity may reduce energy expenditure, thereby allowing them to survive off limited resources and even in severe environments (Storey 2000). For example, hibernating mammals often reduce their basic metabolic rates to as low as 2–4% of their normal rates and maintain their body temperatures within a few degrees above ambient temperatures (Barnes 1989). Similarly, lungfishes (*e.g.*, *Protopterus* sp.) and green-striped burrowing frogs (*Cyclorana alboguttata*) often enter into aestivation to avoid death in summer with water and food restriction (Kayes *et al.* 2009; Navas and Carvalho 2010). Lungfishes hyperventilate and secrete large amounts of mucus which turns into a dry cocoon to conserve energy and retain body water (Ballantyne and Frick 2010), while green-striped burrowing frogs reduce whole animal metabolism by 82%, alongside a reduction in muscle and liver metabolism (Kayes *et al.* 2009).

Nevertheless, prolonged immobilization also triggers a series of problems, such as blood clotting, muscle atrophy, energy shortage, and dysrhythmia, which likely have adverse impacts on animal survival (Boyer and Barnes 1999). Therefore, animals often evolve genetic adaptations to alleviate these adverse effects. For example, Richardson’s ground squirrels (*Spermophilus richardonii*) increase their alpha 2-Macroglobulin at both mRNA and protein levels, which may play an important role in decreasing the coagulative properties of their blood (Srere *et al.* 1995). Genes related to metabolic process, basic cellular process, cellular adhesion, blood coagulation, and immune response showed highly variable expression in Madagascar’s dwarf lemurs (*Cheirogaleus medius*; Faherty *et al.* 2016). Estivating green-striped burrowing frogs are able to regulate the expression of genes in several major cellular pathways critical to the survival and viability of muscle cells while avoiding the deleterious consequences of muscle disuse (Reilly *et al.* 2013). Despite these works, our current knowledge regarding genetic adaptation to prolonged immobilization is largely limited to gene expression profiles with known association to physiological adjustments in a small number of species, particularly mammals.

The Shedao pit-viper (*Gloydius shedaoensis*) presents a unique sedentary lifestyle in extreme seasonal food scarcity. The snakes occur exclusively on the Shedao Island (meaning “snake island” in Chinese), which is a small island off the coast of the Liaodong Peninsula in the Bohai Sea. The island is approximately 0.73 km^2^ in size and 5.3 km away from the nearest continent (Li 1995). The adult snakes are ∼600 mm in length and the generation time is ∼3 years, which is similar to its mainland relative, the black eyebrow pit-viper (*G*. *intermedius*). The census population size of the snake is estimated at approximately 20,000 (Li *et al.* 2007). The most pronounced seasonal change is food availability, although temperature and other climatic variables also change seasonally. The island is an important stopover point for at least 80 species of migratory birds in May-June and August-October every year, which provide the seasonal food resource (*e.g.*, black-tailed gulls *Larus crassirostris*, white waist swifts *Apus pacificus*; Li 1995). The vipers typically remain in hibernation and aestivation without any food or movement for the rest of the year. This may involve corresponding changes in physiology, so as to make the internal environment stable and suitable to the outside environment. Some individuals may fail to obtain food in a year, and fast for up to 18 months (Li 1995). Freshwater is also limited; there are no natural or artificial ponds on the island, and the primary water source for snakes is the dew from the grass and leaves in the morning.

The small size of the island, high population density of snakes, and strong seasonality in food availability together intensify selection pressure on the Shedao pit-vipers. A previous radio-telemetric study indicated that the snakes were extremely sedentary and their average daily displacement was less than 2 m, which may reduce energy expenditure and thus facilitate survival on the food limited island (Shine *et al.* 2003). Therefore, the Shedao pit-viper offers an extreme example to investigate the physiological and genetic mechanisms underlying a sedentary life.

Our objectives are to explore the genetic variations of the Shedao pit-viper that are potentially linked to adaptations to the sedentary life with seasonal food shortage. In particular, we examine 1) whether there is a reduced rate of evolution, as may be expected due to its sedentary lifestyle, and 2) which genes, if any, can be linked to the unique ecology of this island species. We sequenced the transcriptomes of the Shedao pit-viper and its mainland relative, the black eyebrow pit-viper (*G*. *intermedius*). Genomic data of nine other vertebrates were also gathered from public sources to further contextualize island-specific patterns of evolution. Furthermore, we tested genes under positive selection, as well as genes with patterns of convergence between the Shedao pit-viper and other vertebrates with extended hibernation and aestivation.

## Materials and Methods

### Sampling, sequencing, and assembling

One individual of the Shedao pit-viper (*G*. *shedaoensis*) was sampled from the Shedao Island (38° 57′ 0” N, 120° 59′ 0” E) and one individual of the black eyebrow pit-viper (*G*. *intermedius*) was sampled from the Xinbin Manchu Autonomous County (41° 55′ 28” N, 124° 26′ 21”E), Liaoning province, China. These two species are closely related and therefore are appropriate for genetic comparison (Zhao 1980; Shi *et al.* 2016). The two samples were killed with sodium pentobarbital solution and dissected immediately after death. Five tissues, including brain, liver, heart, skeletal muscle, and gonad, were collected. All activities were under permission from local conservation authorities and animal handling followed the approved protocols (protocol number 2017005, Chengdu Institute of Biology).

RNA was extracted separately from each tissue using a standard Trizol protocol (Invitrogen). We mixed the RNA from each tissue in approximately equal quantities for each species. The concentration and integrity of total RNA were examined using agarose gel electrophoresis, a NanoPhotometer spectrophotometer (IMPLEN, CA, USA), as well as an Agilent Bioanalyzer 2100 (Agilent Technologies, CA, USA). RIN scores of the total RNA used for library preparation were greater than 8.6. The NEBNextPoly(A) mRNA Magnetic Isolation Module (NEB, E7490) was used to enrich mRNA. The cDNA libraries were constructed using the NEBNext mRNA Library Prep Master Mix Set for Illumina (NEB, E6110) and the NEBNext Multiplex Oligos for Illumina (NEB, E7500). Insert size was detected by 1.8% agarose gel electrophoresis. Library Quantification Kit-Illumina GA Universal (Kapa, KK4824) was used to carry out a qPCR quantification. The libraries were subsequently sequenced on an Illumina HiSeq2000 platform in Novogene Inc (Beijing, China). Through this process, we obtained approximately 8 Gb raw data of 150bp paired-ends reads for each species. The Q30 of sequencing data were 87.07% and 96.58% for *G*. *shedaoensis* and *G*. *intermedius*, respectively.

We performed quality filtration and *de novo* assembly. The raw reads were first cleaned by filtering out the adapter sequences using Trimmomatic (Bolger *et al.* 2014) with the following parameters: seedMismatches = 2, palindromeClipThreshold = 30, and simpleClipThreshold = 10. High quality reads (>Q20) with less than 10% unknown base calls were retained. The final assemblies were produced using Trinity (Grabherr *et al.* 2011) with default parameters according to the published protocols (Haas *et al.* 2013). Likely open reading frames (ORFs), that were at least 100 amino acids long, were extracted from transcripts in the assemblies using Transdecoder (Haas *et al.* 2013). When multiple transcripts were available for the same genes, only transcripts with the longest CDS were selected for further analyses. The completeness of the transcriptomes of *G*. *shedaoensis* and *G*. *intermedius* was assessed by comparing them to a benchmark set of universal single-copy Tetrapoda orthologs using BUSCO v2 (Simão *et al.* 2015), which includes 3,950 genes.

### Ortholog identification and alignment

Data for nine additional vertebrates were downloaded from the NCBI ftp website (NCBI 2017) or literature-derived website, including the five-pacer viper (*Deinagkistrodon acutus*), the king cobra (*Ophiophagus hannah*), the Burmese python (*Python bivittatus*), the green anole lizard (*Anolis carolinensis*), the American alligator (*Alligator mississippiensis*), chicken (*Gallus gallus*), the western painted turtle (*Chrysemys picta bellii)*, human (*Homo sapiens*), and the western clawed frog (*Xenopus tropicalis*). Detailed assembly version information of these species or data sources are provided in Table S1.

We used the OrthoFinder2 method (Emms and Kelly 2015) to identify putative orthologous groups for the 11 species examined in this study. This method has been demonstrated to be more accurate and faster than other similar methods (Li *et al.* 2003; Emms and Kelly 2015). Prior to the OrthoFinder2 analysis, we first extracted the longest isoform as representative sequence for each gene to generate a nonredundant protein set for each species. We ran OrthoFinder with default parameters using the all-*vs.*-all DIAMOND (Buchfink *et al.* 2015). To construct the repertoire of gene families for each species, the single copy orthologs and paralogs in each orthogroup were assigned into each species. For the single-copy genes, we assigned gene IDs based on annotated genomes. In cases of ID conflict, which were rare, we adopted the majority rule to assign IDs. When conflict occurred for genes of interest, we further blasted the genes against the SwissProt or Nr Database to ensure that correct IDs were assigned. Amino-acid sequences were aligned using MAFFT (v7.427) with its default parameters (Katoh and Standley 2013), and then were converted into corresponding codon alignments using PAL2NAL (Suyama *et al.* 2006). Only single copy orthologs were used for downstream analysis.

### Phylogenomic tree construction

The fourfold degenerate (4D) sites, where all mutations produce synonymous changes, from the concatenated dataset were extracted and used in phylogenomic reconstruction. This was to reduce potential detrimental effects from several confounding factors such as non-phylogenetic signals (Misof *et al.* 2013) and natural selection (Edwards 2009). The concatenated 4D site dataset had 267,162 sites, with 171,047 parsimony informative sites, 71,993 singletons, and 24,122 constant sites. PartitionFinder (Lanfear *et al.* 2012) divided the dataset into 268 subsets of 1,000 sites and determined the best-fit partitioning scheme of these subsets and the optimal model of evolution for each partition, based on a greedy search with RAxML8 (Stamatakis 2014) and the Bayesian Information Criterion (BIC). The partition and model of evolution were subsequently used in a maximum likelihood (ML) analysis. RAxML was used with the best-scoring tree search and a nonparametric bootstrapping (1000 pseudo-replicates).

### Estimation of divergence time and evolutionary rate

We estimated the divergence time between the Shedao pit-viper and the black eyebrow pit-viper using a relaxed molecular clock Bayesian method implemented in the MCMCTREE program (Yang and Rannala 2005; Dos Reis and Yang 2013). Calibration time of each major node was collected from the TimeTree database (http://www.timetree.org/). The specific priors on the calibration nodes are provided in Table S2. Multiple calibration points were used to ensure more realistic divergence time estimates (Donoghue and Benton 2007). The overall substitution rate (rgene gamma) and rate-drift parameter (sigma2 gamma) were set as G (1, 4) and G (1, 4.5), respectively. Trees were generated using the birth-death process with species sampling (birth rate λ, death rate μ, and sampling fraction ρ were set to 1, 1 and 0.1). Additionally, we calculated the absolute rate of molecular evolution based on the 4D sites using the r8s program (Sanderson 2003). The penalized likelihood method and TN algorithm were used to accommodate rate heterogeneity. Furthermore, we calculated the numbers of nonsynonymous and synonymous substitutions for the concatenated data and individual genes from each snake species using the free ratio branch model in the CODEML program implemented in PAML v4.9 (Yang 2007). The difference in dS across genes between the Shedao pit-viper and the black eyebrow pit-viper was examined using the Wilcoxon rank sum test.

### Identification of genes under positive selection (PSGs)

We tested for evidence of positive selection in the Shedao pit-viper through single-copy orthologs using the branch-site model in CODEML, which compared the likelihood of a modified alternative model A (model = 2, NSsites = 2, ω not fixed to 1) and the likelihood of the corresponding null model with ω fixed to 1. The lineage of the Shedao pit-viper was defined as the foreground branch on the species tree. A likelihood ratio test (LRT) was used to determine whether the alternative (selection) model fitted the data significantly better than the null (neutral) model. The false discovery rate (FDR) method was applied to correct for multiple tests. For a gene, if the selection model has a significantly higher likelihood value than the neutral model does (FDR-adjusted *p*-value < 0.05), the gene is considered of having experienced positive selection along the foreground branch. For all genes with evidence of positive selection, we visually inspected and manually corrected their alignments. Alignments containing one or more significantly longer sequences, which created long gaps, were dropped under the assumption that homologous genes should be conserved. Minor adjustments were also applied to some gaps by shifting them to more appropriate locations. All analyses were repeated for the corrected alignments.

### Test for convergent/parallel evolution

Convergent and parallel evolution are often considered evidence of adaptation (Castoe *et al.* 2009), and we examined patterns of convergent and parallel evolution between the Shedao pit-viper and two other vertebrates with extended hibernation and aestivation, the American alligator and the western painted turtle. When two species share the same amino acid residue, if both are independently derived from different ancestral residues, these changes are defined as “convergent”, and if both are independently derived from the same ancestral amino acid residue, these changes are defined as “parallel” (Zhang and Kumar 1997). We tested patterns of convergent and parallel at both the whole genome and individual gene levels. Ancestral sequences reconstruction was carried out using CODEML. The inferred most likely amino acids for the hypothetical common ancestors were extracted from the CODEML output.

For the whole genome level tests, we first calculated the expected number of convergent and/or parallel substitutions based on random chance (the “null”) between every pair of branches using a model-based likelihood method (Castoe *et al.* 2009; Zou and Zhang 2015). The JTT-F_site_ model was used, which provided a relatively high estimate of the null and hence was a conservative estimation of convergent evolution (Zou and Zhang 2015). If the observed numbers of convergent/parallel substitutions are significantly higher than the null, we would conclude a genome wide convergent or parallel evolution. Furthermore, the observed numbers of convergent/parallel substitutions are expected to be positively correlated with the levels of divergence (Castoe *et al.* 2009; Thomas and Hahn. 2015). We plotted the observed numbers of convergent/parallel sites against the number of divergent sites between each pair of branches. The best fit regression line would serve as an empirical null distribution, and we used the distribution to test if there was excessive amount of convergent/parallel sites.

For the individual gene level tests, we conducted two pair-wise comparisons between the Shedao pit-viper and the American alligator and between the Shedao pit-viper and the western painted turtle. All three species have prolonged immobilization and dormancy (Shelton 1934; MacCulloch and Secoy 1983). We first identified amino-acid positions with changes between the two species of each pair. Genes with identified convergent and/or parallel substitutions were subject to Zhang and Kumar’s test (Zhang and Kumar 1997), which calculated the probability that the observed convergent and/or parallel substitutions could be attributed to random chance based on a substitution model. Program converg2 (Zhang and Kumar 1997) was used for this analysis with JTT-F_gene_ model. Similar to PSGs, additional quality control of alignments for genes with convergent/parallel patterns was performed to rule out mis-alignment issues that might generate these patterns.

### Detection of Shedao pit-viper specific amino acid substitution

If the amino acid residues on one site are constant in all species except the Shedao pit-viper, this substitution is defined as a Shedao pit-viper specific amino acid substitution. We used a conserved algorithm implemented in PROVEAN (Choi and Chan 2015) to predict the possible effects of these specific substitutions. Variants predicted as “deleterious” were considered to affect protein functions. To investigate the potential force driving these specific amino acid substitutions, we employed the free ratio branch model in the CODEML program to estimate dN/dS ratios.

### Data availability

The transcriptome data of the Shedao pit-viper (*Gloydius shedaoensis*) and the black eyebrow pit-viper (*G*. *intermedius*), including raw data and assemblies, are available at NCBI (accession number: PRJNA507957). Supplemental material available at figshare: https://doi.org/10.25387/g3.11719890.

## Results

### Assembly and quality assessment

Reads were assembled into 289,801 and 182,076 transcripts for the Shedao pit-viper and the black eyebrow pit-viper, respectively. Contig N50 was 1,470 base pairs (bp) and 1,236 bp, respectively, and detailed assembly statistics are provided in Table 1. Redundancy-minimized datasets were generated by retaining only the longest ORFs for each “gene”. The final data set for downstream analysis contained 31,803 ORFs with an N50 of 1,413 bp for the Shedao pit-viper and 37,320 ORFs with an N50 of 1,032 bp for the black eyebrow pit-viper. BUSCO analysis revealed that the Shedao pit-viper transcriptome contained ∼92% of the 3,950 tetrapod universal single-copy orthologs (full length: 78.6%; partial fragments: 13.4%). Similarly, the black eyebrow pit-viper contained ∼84.2% of the BUSCO single-copy genes (full length: 62.6%; partial fragments: 21.6%). These data suggested that most genes were well assembled.

**Table 1:** Transcriptome assembly information

Species	*Gloydius shedaoensis*	*G. intermedius*
Total number of transcripts	289801	182076
Total number of trinity ’genes’	237412	155080
Total assembled bases	214371261	136100636
Percentage of GC (%)	43.68	45.11
Contig N50 (bp)	1470	1236
Median contig length (bp)	347	396
Average contig length (bp)	739.72	747.49

### Orthogroups inference and alignments

OrthoFinder assigned 213,192 genes (85.9% of total genes from all 11 species considered) to 20,193 orthogroups. Fifty percent of all genes were in orthogroups with 11 or more genes (G50 = 11) and were in the largest 6,823 orthogroups (O50 = 6,823). There were 7,106 orthogroups with all species present and 2,893 of these consisted entirely of single-copy genes. For all downstream analysis, we examined only these single-copy genes. The amino-acid alignments and corresponding codon alignments of these genes were generated, and a concatenated dataset was created from these alignments.

### Phylogenomic tree

**Figure 1:**
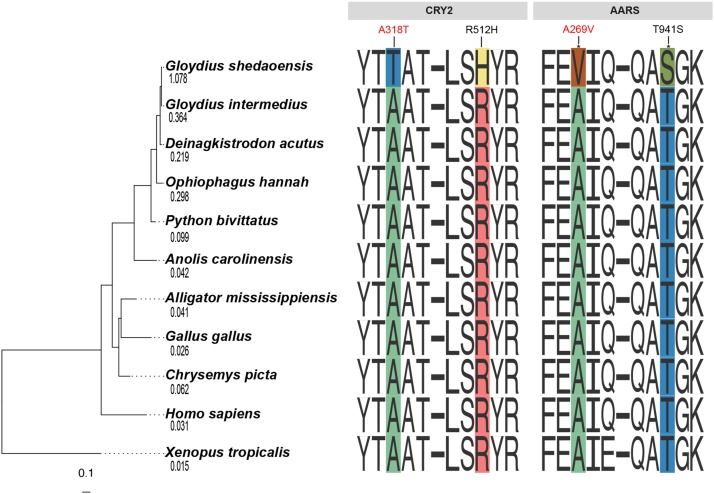
A phylogeny inferred from a maximum likelihood analysis of partitioned fourfold degenerate sites. Numbers under species name are estimated dN/dS ratios for *CRY2*. Four Shedao pit-viper specific amino acid substitutions in the *CRY2* and *AARS* genes are identified. A318T and A269V (in red) are predicted to affect protein functions and A269V and T941S (with asterisks) are under positive selection.

The best-fit partitioning scheme divided the super-matrix into 15 partitions, and the GTR+G model was selected for each subset. The ML analysis resulted in one optimal tree with strong bootstrap support for all interior nodes (100%). The Shedao pit-viper was sister to the black eyebrow pit-viper, and together with the five-pacer viper, they formed a monophyletic group (Figure 1). This species tree is consistent with the well-established vertebrate phylogeny.

### Divergence time and evolutionary rate

The Shedao pit-viper diverged from its mainland relative, the black eyebrow pit-viper, at approximately 2.5 million years ago (MYA; 95% confidence interval: 0.8-6.8 MYA). The Shedao Island was separated from the mainland at approximately one million years ago (Li 1995). The speciation process of the Shedao pit-viper was likely initiated before the formation of the island. All other divergence time estimates are present in supplementary material Figure S1. The estimated absolute rates of nucleotide substitution for all species are provided in Table 2. The Shedao pit-viper had a rate of 9.68E-04 substitutions per site per million years, which was much lower than its mainland relative, the black eyebrow pit-viper (1.40E-03). The substitution rate of the Shedao pit-viper was similar to that of the American alligator (8.23E-04), which also has prolonged hibernation, suggesting that metabolic suppression induced by long-term inactivity might have caused the rate reduction in the Shedao pit-viper. In addition, for the concatenated data set, the numbers of non-synonymous (dN: 0.00038) and synonymous substitutions (dS: 0.00243) per site and the dN/dS ratio (0.165) of the Shedao pit-viper were also much lower than those of the black eyebrow pit-viper (0.00189, 0.00485, and 0.390, respectively). The numbers of dS across genes in the Shedao pit-viper were significantly lower than these in black eyebrow pit-viper (Wilcoxon rank sum test, W = 110794, *P* = 0.0187; Wilcoxon 1945).

**Table 2:** The estimated nucleotide substitution rates of the11 species examined in this study. The rate unit is number of substitutions per site per million years

Species	Evolutionary Rate
*Chrysemys picta*	5.70E-04
*Deinagkistrodon acutus*	7.00E-04
*Python bivittatus*	7.58E-04
*Alligator mississippiensis*	8.23E-04
*Gloydius shedaoensis*	9.68E-04
*Gloydius intermedius*	1.40E-03
*Ophiophagus hannah*	1.48E-03
*Gallus gallus*	1.62E-03
*Anolis carolinensis*	1.73E-03
*Xenopus tropicalis*	1.74E-03
*Homo sapiens*	1.91E-03

### Genes under positive selection

We initially detected two genes (*SERPINC1* and *AARS*) with evidence of positive selection (PSGs) along the Shedao pit-viper lineage (Table S3 and File S1). Only *SERPINC1* (Serine Proteinase Inhibitor, Clade C, Member 1) remained significant after adjustment for multiple tests using FDR (adjust p-value∼0), while *AARS* was above the significant threshold (Table S3). The detection power was low since many comparisons were made. Both genes had two unique amino acid substitutions (D38A and R438D for SERPINC1; A269V and T941S for AARS) that were likely associated with the positive selection signals in the Shedao pit viper. *SERPINC1* is the most important serine protease inhibitor in plasma that regulates the blood coagulation cascade (Szabo *et al.* 2005). *AARS* (Alanyl-TRNA Synthetase) encodes alanyl-tRNA synthetase, and mutations in *AARS* may lead to muscle weakness and atrophy (Latour *et al.* 2010).

### Convergent and parallel evolution

**Figure 2:**
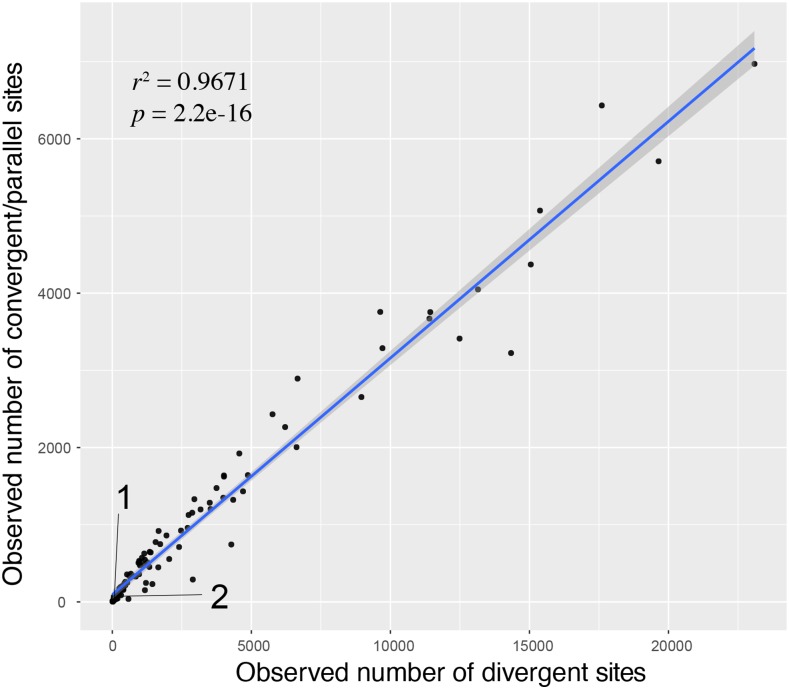
Correlation between observed number of divergence sites and observed number of convergent and parallel sites. Each data point represents one pairwise comparison between two branches, including both exterior and interior branches. Data point 1 = the Shedao pit-viper *vs.* the western painted turtle; data point 2 = the Shedao pit-viper *vs.* the American alligator.

We did not detect a genome-wide pattern of convergent or parallel evolution. In all cases, the observed numbers of convergent and parallel substitutions for all pairwise comparisons were smaller than the expected numbers under random expectation. Furthermore, we detected a significant positive correlation between the number of convergent/parallel substitutions and the number of divergent substitutions as expected (*r^2^* = 0.9671, *P* < 2.2e-16; Figure 2). The observed numbers between the three species with extended hibernation and aestivation were expected at their levels divergence, and no excessive convergent and/or parallel substitutions were detected (Figure 2).

At the individual genes level, 18 genes were identified as having convergent or parallel substitutions between the Shedao pit-viper and the western painted turtle or between the Shedao pit-viper and the American alligator (Table S4, S5 and File S2). Among them, the convergent substitutions in three genes and parallel substitution in 13 genes were significantly more than random chance events (Zhang and Kumar’s test; *P* < 0.05). *KLF10* (Kruppel Like Factor 10) was a convergent gene between the Shedao pit-viper and the American alligator (*P* = 0.001261). It is a transcriptional repressor and regulates the circadian clock (Blok *et al.* 1995). *ACAT1* (Acetyl-CoA acetyltransferase 1) possessed both convergent and parallel substitutions between the Shedao pit-viper and the American alligator. It encodes an enzymes that catalyzes the last step of the fatty acid beta-oxidation pathway (Fukao *et al.* 1991). *PRSS35* (Serine Protease 35), a critical ovarian protease conserved throughout the course of vertebrate evolution, possessed parallel substitutions between the Shedao pit-viper and the American alligator (*P* = 0.043295), as well as between the Shedao pit-viper and the western painted turtle (*P* = 0.04934) at the same amino acid site (Table S5).

### Shedao pit-viper specific amino acid substitutions

Two specific amino acid substitutions were observed in the *CRY2* gene (A318T and R512H) in the Shedao pit-viper (File S3). *CRY2* (Cryptochrome Circadian Regulator 2) acts as transcription repressors within the circadian clockwork (Reppert and Weaver 2002) and plays a pivotal role in the generation and maintenance of circadian rhythms (Klarsfeld *et al.* 2004). The black eyebrow pit-viper maintained the same amino acid residues as all other species examined in this study. A total of 182 sequences of *CRY2* in PROVEAN were used to generate prediction of functional effects of those Shedao pit-viper specific amino acid substitutions. The A318T variant was predicted to have a “deleterious” effect with a PROVEAN score of -3.009 (the predefined threshold = -2.5). The R512H variant was predicted to have a “neutral” effect with a score of -2.053 (Figure 1). In addition, free ratio model presented the highest value of dN/dS along the Shedao pit-viper branch (1.078; Figure 1) for *CRY2*, implying positive selection or relaxed negative selection have likely driven the evolution of *CRY2* in this species with prolonged dormancy.

The *AARS* gene, which is also under positive selection, had two Shedao pit-viper specific amino acid substitutions (A269V and T941S). *AARS* is associated with muscle weakness and atrophy (Latour *et al.* 2010), and interestingly, the two substitutions were identified as under positive selection by PAML branch-site models with Bayes Empirical Bayes (BEB) analysis. In addition, the A269V variant was predicted to have a “deleterious effect” on protein function in PROVEAN (Figure 1). A third gene, *MKKS* (McKusick-Kaufman Syndrome), also contained two specific amino acid substitutions (S2F and S58A) and S2F likely had a “deleterious effect”. The gene acts as a chaperonin that helps in folding other proteins. The encoded protein may play an important role in the formation of limbs, heart, and reproductive system, and mutations in this gene may result in congenital heart defects (Scott *et al.* 2017).

## Discussion

Here we investigated the sedentary lifestyle of the Shedao pit-viper with extreme food seasonality and detected several important and interesting genetic patterns. The evolutionary rate of the Shedao pit-viper is greatly reduced compared to its mainland relative, the black eyebrow pit-viper. Two genes (*SERPINC1* and *AARS*) bear signals of positive selection along the Shedao pit-viper lineage, and 16 genes (including *ACAT1*, *KLF10*, and *PRSS35*) present convergent and/or parallel substitutions between the Shedao pit-viper and two other inactive vertebrates, the western painted turtle and the American alligator. In addition, *CRY2* and *AARS* genes have several Shedao pit-viper specific amino acid substitutions. Those patterns corroborated with each other and advanced our understanding of the genetic basis of several critical physiological traits related to the extreme sedentary lifestyle, including anti-thrombosis, energy derivation, circadian rhythm, and retention of muscle tone.

The Shedao pit-viper exhibits a much lower substitution rate relative to its mainland relative; this is possibly a consequence of its low metabolic rate and long period of immobilization. A previous radio-telemetric monitoring study indicated that their average daily displacement distance is less than 2 m (Shine *et al.* 2003). In comparison, other viperid species display movements of > 10 m/day and ranges of > 10^4^ m^2^ (Macartney *et al.* 1988) and some rattlesnakes may have ranges up to 2 × 10^6^ m^2^ (Reinert and Zappalorti 1988). Variations in nucleotide substitution rates are strongly correlated with body size, metabolic rate (Martin and Palumbi 1993), and generation time (Laird *et al.* 1969). Given the similar body size and generation time for the Shedao pit-viper and its sister species (Zhao 1980), the substantial difference in nucleotide substitution rates is likely caused by the depression in metabolic rate in the Shedao pit-viper (Donoghue and Benton 2007; Liu 2008). Lower metabolic rate (and therefore lower rates of oxygen radical flux) reduces oxidative damage to DNA (Shigenaga *et al.* 1989; Fraga *et al.* 1990), which in turn decreases rate of DNA synthesis, cuts down errors of replication, depresses frequency of repair (Martin and Palumbi 1993). All these processes lead to low rate of nucleotide substitution. Not surprisingly, the American alligator and the western painted turtle, which have extended immobilization and hibernation (Shelton 1934; MacCulloch and Secoy 1983), also showed low rates of neutral evolution (Table 2).

Several genes with evidence of positive selection, patterns of convergence or parallelism, or Shedao pit-viper specific amino acid substitutions, including *SERPINC1*, *ACAT1*, *KLF10*, *PRSS35*, *CRY2*, *MKKS*, and *AARS*, likely play important roles in the adaptation process of the Shedao pit-vipers to their sedentary lifestyle on the island. Extended immobilization may expose organisms to diverse stressors, including increased risk of thrombosis, energy deficiency, and muscle atrophy (van Breukelen *et al.* 2010). Substitutions in these genes may improve functions related to thrombosis and energy utilization, alleviate muscle atrophy, and facilitate the Shedao pit-vipers to cope with those adverse effects. For example, one gene under positive selection has function related to thrombosis. The protein coded by *SERPINC1* inhibits thrombin as well as other activated serine proteases of the coagulation system, including Thrombin, Factor Xa and Factor IXa (Bedsted *et al.* 2003). Mutations of *SERPINC1* in the Shedao pit-viper may increase antithrombin activity and antithrombin concentration in the blood, which can prevent formation of blood clots during extended immobilization.

The *AARS* gene involves muscle function. It contains two Shedao pit-viper specific amino acid substitutions, and both bear signals of positive selection and one variant likely impacts protein function. As a pathogenic gene, mutations in *AARS* may cause Charcot-Marie-Tooth disease 2N (CMT2N), characterized by progressive weakness and atrophy (Latour *et al.* 2010), and early infantile epileptic encephalopathy-29 (EIEE29), characterized by refractory seizures, neurodevelopmental impairment, and poor prognosis (Simons *et al.* 2015). The observed substitutions in *AARS* may minimize degradative pathways of muscle atrophy and help Shedao pit-vipers to preserve their muscle functions.

Several genes involve internal clock and rhythm. As a core component of internal time-keeping system, the *CRY2* gene has two Shedao pit-viper specific amino acid substitutions. *CRY2* plays a photoreceptive role; it also regulates various physiological processes through sustaining an approximately 24 hr circadian rhythms in gene expression and acting on the CLOCK:BMAL1 heterodimer, which modulates rhythms in metabolism and behavior (Vitaterna *et al.* 1999; Klarsfeld *et al.* 2004). Circadian rhythms interact with the environment and help individual to maintain a harmonious relationship with environment. *CRY2* mutants may alter circadian rhythms and stop mRNA cycling (Emery *et al.* 1998). These Shedao pit-viper specific substitutions in *CRY2* could be an adaptive consequence of lone-term sedentary life on island. The *KLF10* gene, which has convergent substitutions between the Shedao pit-viper and the American alligator, binds to the promoter of the core clock component ARTNL/BMAL1 and plays a role in the regulation of the circadian clock. It is involved in the process of cellular response to starvation by regulating the circadian expression of genes involved in lipogenesis, gluconeogenesis, and glycolysis in liver (Blok *et al.* 1995). It is a link between the circadian clock and metabolism in liver, which is important for the Shedao pit-viper to cope with the scarce food resource on island.

An energy derivation related gene, *ACAT1* has one convergent substitution between the Shedao pit-viper and the American alligator. The encoded protein is associated with a key catalytic step of mitochondrial beta-oxidation pathway, an important part of energy supply. The Shedao pit-vipers feed exclusively on migratory birds in spring and fall, and they often fast for months (Li 1991). Modifications in fatty acid beta-oxidation process likely help Shedao pit-vipers to survive the island life.

## Conclusion

Our analyses revealed several important genetic signals in the Shedao pit-viper that likely represent adaptation to an extreme sedentary lifestyle with seasonal food availability. Positive selection was detected in two genes involved in antithrombin (*SERPINC1*) and muscle atrophy (*AARS*). Multiple Shedao pit-viper specific amino acid substitutions were observed in core clock component *CRY2* and *AARS*. Convergent and parallel substitutions were found in a fatty acid beta-oxidation related gene (*ACATA1*) and circadian gene (*KLF10*). We also detected a low rate of evolution in the Shedao pit-viper. These genetic signals suggest that critical physiological traits related to thrombosis, energy source, circadian clock, and muscles atrophy are important in the adaptation process to the sedentary life of the Shedao pit-viper. Future research along these directions should be fruitful.
